# Spatial Distribution of Toxic Metal(loid)s and Microbial Community Analysis in Soil Vertical Profile at an Abandoned Nonferrous Metal Smelting Site

**DOI:** 10.3390/ijerph17197101

**Published:** 2020-09-28

**Authors:** Jiejie Yang, Siqi Wang, Ziwen Guo, Yan Deng, Menglong Xu, Siyuan Zhang, Huaqun Yin, Yili Liang, Hongwei Liu, Bo Miao, Delong Meng, Xueduan Liu, Luhua Jiang

**Affiliations:** 1 School of Minerals Processing and Bioengineering, Central South University, Changsha 410083, China; jiejieyang@csu.edu.cn (J.Y.); wangsiqi97@csu.edu.cn (S.W.); guoziwen@csu.edu.cn (Z.G.); dengyan@csu.edu.cn (Y.D.); xumenglong@csu.edu.cn (M.X.); siyuanzhang.bio@csu.edu.cn (S.Z.); yinhuaqun@csu.edu.cn (H.Y.); liangyili6@csu.edu.cn (Y.L.); hongweiliu@csu.edu.cn (H.L.); miaobo@csu.edu.cn (B.M.); delong.meng@gmail.com (D.M.); xueduanliu@csu.edu.cn (X.L.); 2 Key Laboratory of Biometallurgy of Ministry of Education, Central South University, Changsha 410083, China

**Keywords:** metal(loid)s pollution, smelting site, soils properties, microbial community analysis

## Abstract

In this study soils at different depths were collected in a Zn smelting site located in Zhuzhou City, China, in order to understand toxic metal(loid)s distribution and microbial community in vertical soil profile at a smelting site. Except Soil properties and metal(loid)s content, the richness and diversity of microbial communities in soil samples were analyzed via high-throughput Illumina sequencing of 16s rRNA gene amplicons. The results showed that the content of As, Pb, Cu, Cd, Zn, and Mn was relatively high in top soil in comparison to subsoil, while the concentration of Cr in subsoil was comparable with that in top soil due to its relative high background value in this soil layer. The bioavailability of Cd, Mn, Zn, and Pb was relative higher than that of As, Cr, and Cu. The diversity of soil microbial communities decreased with increasing depth, which might be ascribed to the decrease in evenness with increase in depth duo to the influence by environmental conditions, such as pH, TK (total potassium), CEC (cation exchange capacity), ORP (oxidation reduction potential), and Bio-Cu (bioavailable copper). The results also found *Acidobacteria*, *Proteobacteria*, *Firmicutes*, and *Chloroflexi* were dominant phyla in soil samples. At the genus level, *Acinetobacter*, *Pseudomonas*, and *Gp7* were dominant soil microorganism. Besides, Environmental factors, such as SOM (soil organic matter), pH, Bio-Cu, Bio-Cd (bioavailable cadmium), and Bio-Pb (bioavailable lead), greatly impacted microbial community in surface soil (1–3 m), while ORP, TK, and AN concentration influenced microbial community in the subsoil (4–10 m).

## 1. Introduction

As a critical component for terrestrial ecosystems, soil plays a crucial role in the survival of living beings through providing habitats and nutrient resources [1,2]. However, with rapid industrialization over the last century, a series of anthropogenic activities have led to the release potential toxic metal(loid)s into the soil ecosystem due to the lack of environmental management and monitoring [3]. 16.1% of soil is contaminated in China and inorganic pollutants, such as Hg, Cd, As, Cu, Cr, Pb, Zn, and Ni are found in high quantities in 82.8% soil of contaminated sites, as reported by the China Soil Pollution Survey published in 2014 [4]. Among anthropogenic activities, the nonferrous metal smelting is one of the main sources contributing the potential toxic metal(loid)s pollution on the soil, especially in central south and southwest regions of China [5]. During the extraction of metal from mineral ores, a large number of slags were deposited on the dumps at some historical smelting sites. After weathering and rainwater leaching, the irregular disposed slags inevitably released metal(loid)s into the soil, which resulted in soil contamination and threating the health of the soil ecosystem and nearby habitats [6,7].

Recently, many studies have mainly been conducted on spatial distribution and risk assessment of metal(loid)s in polluted soil [8,9,10,11]. For instance, Qu et al. investigated the spatial distribution of metal(loid)s and assessed their potential ecological risks in urban soils [12]. Wang et al. examined metal(loid)s spatial distribution in soils that were collected from a typical industrial area and also evaluated their ecological risk [13]. In addition, metal(loid)s are generally toxic to microorganisms [14]. Thus, the existence of high concentration of metal(loid) level in contaminated soils will influence the microbial communities because of its toxicity ability. There have been reports that metal(loid)s could affect various microbiological vital functions in soil, including growth, morphology, adhesion and biochemical activities, respiration, and nitrogen and phosphorus geochemical cycles, therefore resulting in a reduction in microbial diversity and changes in microbial community structure [3,15]. Based on this, previous researches, by using fingerprint methods, have revealed that metal(loid)s pollution had obvious impacts on soil microbial communities either in a short-term or long-term period [16]. Nevertheless, the fingerprint techniques can only provide limited information on microbial communities [16]. A more comprehensive study on microbial diversity and the abundance of microbes in soil has recently been obtained using high-throughput sequencing methods, i.e., Illumina sequencing of 16S rRNA gene amplicons, due to their access to more information on the phylogenetic compositions of microbial communities in soil [3,17]. For example, some studies have obviously shown that the investigation in microbial diversity via the high-throughput methods is 100 times more in comparison to the traditional culture-dependent techniques [15]. Accordingly, studying the richness and diversity of microbes based on the 16S rRNA gene amplicons sequencing method will be helpful in offering significant information to soil risk management and bioremediation of the metal(loid)s polluted areas.

Keeping these in view, research on the spatial distribution of potential toxic metal(loid)s in soil, as well as its effects on microbial communities, was conducted at an abandoned nonferrous smelting site in Zhuzhou City, Central-South China. Specifically, soil samples from different soil profile in the smelting site were collected in order to examine their properties and metal(loid)s distribution. In addition, microbial communities in the metal(loid)s polluted soil were also investigated through the Illumina MiSeq sequencing (Illumina, San Diego, CA, USA) to analyze the microbial composition and diversity. Moreover, the influences of soil characteristics and metal(loid)s pollutant on the soil microbial communities were further explored.

## 2. Materials and Methods

### 2.1. Study Area and Soil Sampling

**Figure 1:**
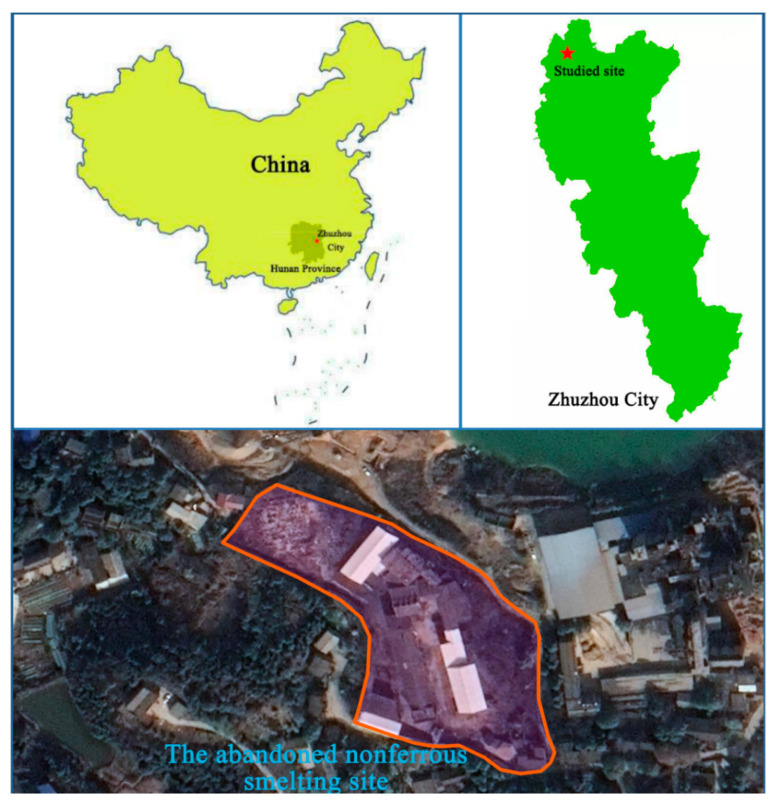
Location of the studied site.

The abandoned nonferrous smelting site with a total area of 22294.65 m^2^ is situated in Zhuzhou City, Hunan Province, China (coordinates 27°52′51.4′′ N and 113°4′15.6′′ E), as shown in Figure 1. This smeltery discontinued in 2011 was mainly engaged in the production of zinc oxide using zinc-containing waste residue through pyrometallurgy. The south and west of the site is adjacent to residential areas and the east and northeast of the site is adjacent to a cement plant. In this study, soil samples in different section of soil profile were collected in June, 2019 from three holes, as shown in Figure S1 (Supplementary Material). Soil samples at 1–10 m in depth were obtained by a percussion drilling from the location in the central area of the nonferrous smelting site, because this area is close to the manufacturing shop, which might contaminated by high concentration of metal(loid)s. The background sample was collected in the northwest of the site, where no human disturbance was found and percussion drilling worked until struck a hard rock layer in the soil. The obtained soil cores were divided in 1 m slices and then preserved in polyethylene bags respectively and immediately brought back to our laboratory. After removing large particles of slag and stones, the soil samples utilized to microbial analyses were stored in −80 °C refrigerator and the samples used to physicochemical examination were air-dried and ground to pass through a 100-mesh sieve and then stored at 4 °C refrigerator.

### 2.2. Soil Properties and Metal(loid)s Determination

The moisture content (MC) of soil is represented as the ratio of the mass of water held in the soil to the dry soil. The mass of water was examined by the difference before and after drying the soil in 104°C for 24 h [18]. The soil pH and oxidation reduction potential (ORP) were measured under a soil: water ratio of 1:2.5 (wt/vol) by a digital pH/ORP meter with a glass electrode and an Ag/AgCl reference electrode (BPH–220, Bell Instrument Equipment Co. Ltd., Dalian, China), respectively [19]. The contents of ammonia nitrogen (AN) was examined by shaking 10 g of air-dried soil in 100 mL of 2 M KCl for 1 h and then recorded by indophenol blue method. Nitrate nitrogen (NN) was analyzed by shaking 5 g of air-dried soil in 50 mL of 2 M KCl for 1 h and then determined by ultraviolet spectrophotometry (UV–2550, Shimadzu, Kyoto, Japan). The available phosphorus (AP) was extracted by NaHCO_3_ in soil and then analyzed through using Mo–Sb antispetrophotography method. The soil total potassium (TK) was determined by the NaOH fusion method and then recorded by atomic absorption spectroscopy (GGK-830, Haiguang Instrument, Beijing, China); available potassium (AP) was extracted through shaking 1 g of air-dried soil in 10 mL of 1 M NH_4_OAc for 5 min.; after being filtered, the filtrate was also determined by atomic absorption spectroscopy (GGK-830, Haiguang Instrument, Beijing, China). The cation exchange capacity (CEC) in soil was examined by the spectrophotometry (UV–1800PC, Mapada Instruments, Shanghai, China) with hexaamminecobalt(III) chloride as exchange ion [20]. The soil organic matter (SOM) was analyzed by using potassium dichromate oxidation-ammonium ferrous sulfate titration. Soil texture was investigated via hydrometer and sieve, in which the grain size groups were determined: clay (<0.002 mm), silt (0.002–0.02 mm), and sand (0.02–2 mm). The total metal concentrations were determined, as follows: after being ground to pass through 100 mesh sieves, 0.50 g soil sample was accurately weighed into polytetrafluoroethylene tube and then 10 mL HNO_3_, 5 mL HF, and 2 mL HClO_4_ was added with shaking 60 s. After that, the samples were placed on the electric heating plate (XJS20–42, Laboratory Instrument Equipment Co., Ltd., Tianjin, China) in ordinary pressure. In the temperature procedure, the temperature was set of 110 °C for 30 min., 140 °C for 30 min., and 180 °C until soil was completely digested and obtained clarified digestion fluid. Subsequently, the supernatant in the tube was transferred to 25 mL volumetric flask. Finally, the concentration of metal(loid)s (As, Pb, Cd, Cu, Zn, Cr, and Mn) in the supernatant was examined via inductively coupled plasma atomic emission spectrometry (ICP–AES) [8]. Reagent blanks and standard reference materials (GBW07430) were used in each batch of digestion to guarantee the relative standard deviations (<5%) in order to control the quality of the analytical method. Besides, the three-step sequential extraction procedure was conducted based on the modified BCR; and, the metal(loid)s was divided into four fractions in this procedure, including weak acid soluble fraction, reducible fraction, oxidizable fraction, and residual fraction [21]. In this study, all of the physicochemical parameters were analyzed in triplicate.

### 2.3. DNA Extraction, PCR Amplification, and High Throughput Sequencing

The genomic DNA in soil was extracted by Fast DNA Spin Kit for Soil (MP Biomedicals Inc., California, USA) based on the manufacturer’s protocol. Polymerase chain reaction (PCR) in V4 region was conducted at the Applied Biosystems 2720 Thermal Cycler (ABI Inc., Waltham, MA, USA) via using primer pair 515F (5′-GTGCCAGCMGCCGCGGTAA-3′) and 806R (5′-GGACTACHVGGGTWTCTAAT-3′) in order to amplify the 16s rRNA gene of bacteria and archaea. The PCR system (25 μL) included 2 μL of template DNA, 0.5 μL (10 nM) of each primer, 12.5 μL of 2 × Taq PCR Master Mix (Vazyme, Piscataway, NJ, USA), and 9.5 μL of DNase-Free deionized water. PCR procedure was set, as follows: an initial denaturation was set at 94 °C for 5 min. followed by 30 cycles of 94 °C for 20 s, 55 °C for 25 s annealing and 68 °C for 45 s extension, with final extension at 68 °C for 10 min. The PCR products were subjected to agarose gel electrophoresis for recovery through E.Z.N.A. TM Gel Extraction Kit (OMEGA Biotek Inc., Doraville, GA, USA). After that, the quality of recovered PCR products was examined by using Nanodrop Spectrophotometer (ND-1000 Spectrophotometer, Nanodrop products, Wilmington, DE, USA). Finally, the sequencing library was constructed using the mixture of each recovered PCR product (150 ng). The Miseq 500 cycles kit was used to 2 × 250 bp paired-ends sequencing on Illumina MiSeq sequencing platform (Illumina, San Diego, CA, USA) [19]. In each soil depth, triplicate samples were determined and 90 samples were analyzed. In above process, all of the tubes and weighing scoops that were used in weighing samples were sterile and disinfected with alcohol in order to ensure no cross contamination between samples. Experimental tools, such as centrifuge tube spear and suction head of pipette used in the extraction of soil DNA samples, were sterilized by high-pressure steam at 101 kPa and 121 °C. The suction head of pipette was also replaced after used. Besides, the PCR procedure was performed on a super-clean bench, and sequencing was also performed in a sterile room, and the operation was performed in strict accordance with the aseptic operation standards.

### 2.4. Data Process and Statistical Analyses

Amplicon libraries were constructed using the VAHTSTM Small RNA Library Prep Kit for Illumina (Vazyme, Nanjing, China) and adding the adapter as identification tags between different libraries. Double-end sequencing of 2 × 250 bp cycle was performed on the library using Miseq 500 cycle kit (Illumina, San Diego, CA, USA). Approximately, 2.5 GB raw reads were generated after Illumina sequencing. The sequencing data were merged, trimmed, filtered, aligned, and clustered by the Galaxy pipeline that was developed by Prof. Zhou’s lab (http://zhoulab5.rccc.ou.edu/) at University of Oklahoma. Sequences with 97% identity were assigned to the same OUT using UPARSE. Taxonomic assignment was performed using RDP classifier (http://rdp.cme. msu.edu/classifier/classifier.jsp). When the similarity was lower than 50%, the unit was assigned to unclassified. The samples were resampled to the 11,000-sequence depth in order to eliminate the impact led by different sequencing depth.

Shannon diversity index, Simpson’s index of diversity, Pielou evenness index, Chao1 index, Detrended Correspondence Analysis (DCA), Canonical Correspondence Analysis (CCA), Non-metric Multidimensional Scaling (NMDS), and Analysis of Similarities (ANOSIM) were calculated by using the base R package vegan (v. 2.5.6). The Venn diagram was plotted using ‘VennDiagram’ Package. The linear discriminant analysis effect size (LEfSe) statistical analysis was conducted at the online interface Galaxy (http://huttenhower.sph.harvard.edu/galaxy) with an alpha value < 0.05 and a linear discriminant analysis score > 2. The genera with relative abundance greater than 1.5% were selected for analysis. Pearson correlation analyses between microbial and environmental variables were calculated by using Minitab 17.0 software (Minitab Inc., State College, PA, USA). The Mean and the Standard Deviation were calculated using SPSS 20.0 software (SPSS Statistics 20, IBM, Armonk, NY, USA). The graph of relative abundance and rarefaction curve was plotted by Origin 2020b (OriginLab, Northampton, MA, USA). The graph of CCA was plotted by R package ggplot2 (v. 3.2.1).

## 3. Results and Discussion

### 3.1. Soils Characteristics and Vertical Distribution of Metal(loid)s

**Figure 2:**
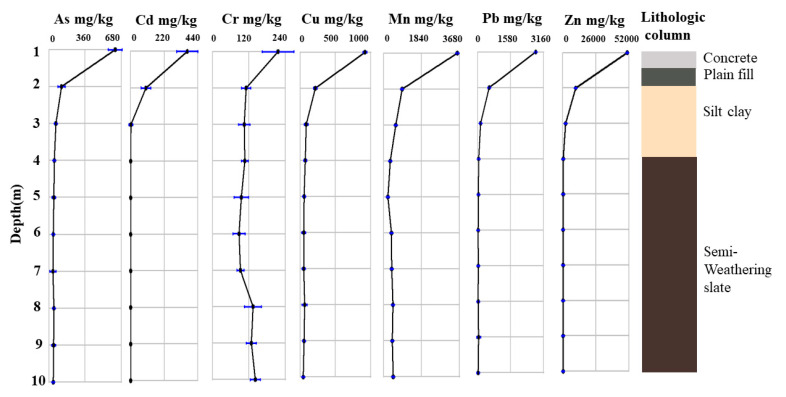
The mean concentration of As, Cd, Cr, Cu, Mn, Pb and Zn in soil vertical profile.

Table 1 shows the soil properties, including pH, ORP, granulometry, MC, SOM, AN, NN, AP, AK, TK, and CEC in different depth of contaminated soils. As seen, soil deposited in the top layer (0–1 m) is mainly composed of sand (the content of 54.3%). The layer (2–3 m) of polluted soil had the highest content of clay fraction contained about 44.9–51.6% of this fraction. In the subsoil (4–10 m), silt fraction contained about 45.1–54.2% (the highest value). The granulometry results were consistent with the lithology of the contaminated soil (0–10 m), as reflected in Figure 2. the main components in the soil depth of 0–1 m was concrete and plain fill; in 2–3 m the main components were silty clay and in 4–10 m was semi-weathering slate. The soil pH values in different depth varied from 4.5 (7 m) to 7.5 (1 m), indicating the soil as neutralalkaline in 1–3 m and acidic in 4–10 m, as listed in Table 1. Higher pH values were obtained in the upper layer of soil possibly due to the effect of slags which contained alkaline minerals; and the lower pH values in the subsoil might ascribe to the acid red soil [22]. Besides, the ORP in different depth of soil was generally less than 300 mV, which mainly contributed to the anaerobic environment of soil [23]. The MC values in soil were about 20% and the values in top layer was slightly larger than the lower soil layer. The layer in 0–3 m of soil showed several times higher CEC values than the lower soil layer (4–10 m). The SOM content showed a range of 0.3–6.5%. However, soil at depths of 1 m and 2 m exhibited relatively higher values, i.e., 6.5% and 2.2%, respectively. The contents of AP and AK in the upper soil were significantly higher than those in the lower soil. The contents of AN in soil vertical profile greatly fluctuated, possibly due to the denitrification under anaerobic conditions in subsoil [24].

**Figure 3:**
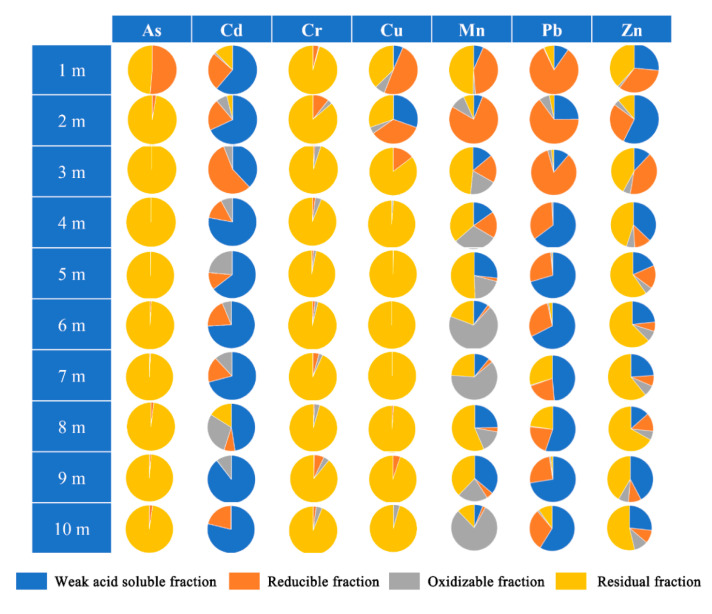
Compositions of four fractions for each metal(loid) in different soil depths.

Figure 2 plots the mean concentration of As, Cd, Zn, Pb, Cu, Cr, and Mn in the vertical soil profile. The concentration ranges of metal(loid)s in different depth of soil were: 37.3–619.7 mg/kg for As, 0–368.8 mg/kg for Cd, 88.3–215.0 mg/kg for Cr, 45.4–918.6 mg/kg for Cu, 222.5–3392.0 mg/kg for Mn, 25.3–2767.7 mg/kg for Pb, and 97.4–50541 mg/kg for Zn. Besides, the background values of metal(loid)s at this site for As, Cd, Pb, Cr, Cu are rather low, while these values for Mn and Zn are comparative high, as reflected by Figure S2. Mn, Zn, Pb, and Cu were abundant in the studied soil. The high concentrations of the majority of metal(loid)s in the vertical profile were found mainly in 1–2 m of soil. However, the concentration of Mn was maintained at a high level, even at a depth of 10 m with concentration of 510 mg/kg, and the concentrations of Cr in 3–10 m were comparable with that in top soil. This might be ascribed to its relative high background value in the subsoil, as shown in Figure S2. As, Cd Pb, and Mn were the risk pollutants in the soil vertical profile, according to the risk control standard for soil contamination of development land. The concentration of As in 1–2 m exceed the risk intervention values for soil pollution of first type development land (120 mg/kg) and 3–10 m was above the risk screening values for soil pollution of first type development land (20 mg/kg); for Cd, only the concentration in 1–2 m exceeded the risk intervention values for soil pollution of first type development land (47 mg/kg); the concentrations of Pb were high at a depth of 1 m (2765.7 mg/kg), exceeding the risk intervention values (800 mg/kg) [25]. Four metal(loid)s’ fractions were also analyzed by using modified BCR, as shown in Figure 3. As seen, residual fraction was the predominant in all samples of As and Cr; and reducible fraction of As only significantly enriched at 1 m depth. Metal(loid)s in residual fraction were tightly bound to the crystalline structures of the minerals that existed in the soil matrices. This suggested that Cr and As are less likely to be released. Besides, 80% Cr existed as Cr(III) and 70% As presented as As(V), as shown in speciation distributions of As and Cr (Figure S3). This indicated that the toxicity to soil microorganisms of Cr and As was very small. For Cu, the weak acid soluble fraction slightly occurred at 1–2 m depth and residual fraction was the predominant in other samples at 3–10 m depth. This suggested that the mobility of Cu was comparative high at 1–2 m depth. In addition, the fractions of Cd, Mn, Pb, and Zn were distributed unevenly at different depth. As a whole, the weak acid soluble fraction accounted for a large proportion. This indicated that Cd, Mn, Pb, and Zn in the soils of this site might lead to a major ecological risk, because of the ease with which they might be leached in slightly acidic, or neutral conditions, followed by assimilation by soil organisms. Chemical fractionation analysis can also provide information regarding the bioavailability of metal(loid)s. The first step of the BCR was considered as bioavailable fraction, which was likely ready to be absorbed by organism [26]. Table S1 summarizes the bioavailable forms of different metal(loid)s in various soil depths.

### 3.2. Alpha-Diversity and Composition of Archaea and Bacterial Communities

**Figure 4:**
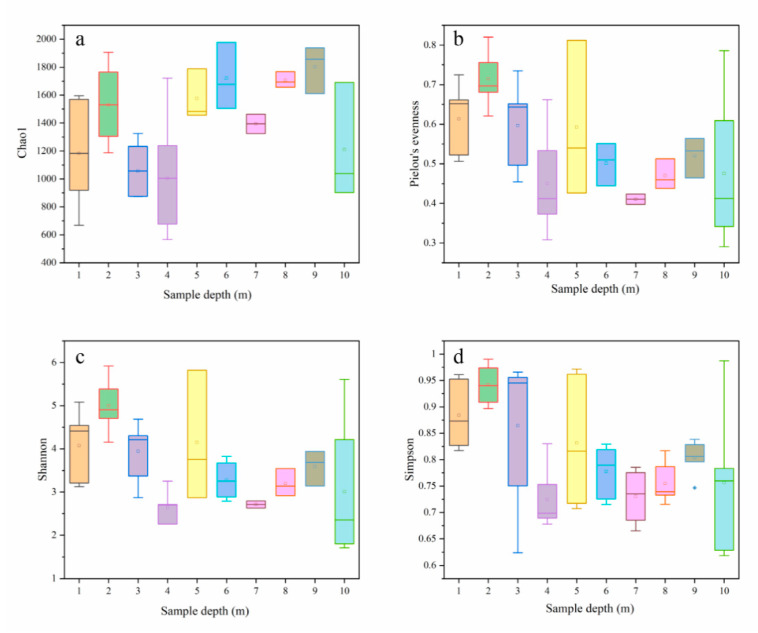
Chao1 index (**a**), Pielou’s evenness index (**b**), Shannon’s diversity index (**c**), and Simpson’s index of diversity (**d**) in soil at different depths.

**Figure 5:**
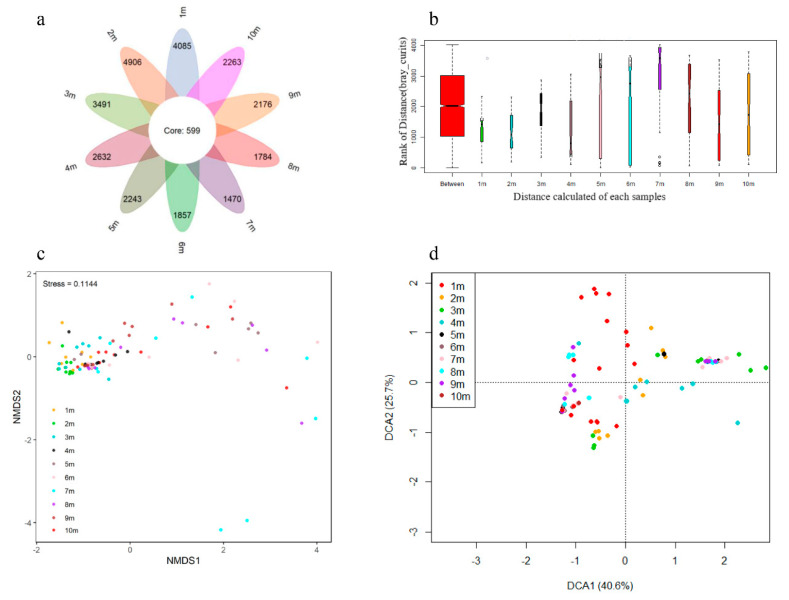
Venn diagram (**a**), Analysis of Similarities (ANOSIM) (**b**), Non-metric Multidimensional Scaling (NMDS) (**c**), and Detrended Correspondence Analysis (DCA) (**d**) analysis in OUT among ten depth layers of soil.

A total of 1,129,918 quality 16s rRNA gene sequence reads were obtained from the high-throughput sequencing for soil samples at different depths, which could be clustered into 8217 OTUs. The rarefaction curve, as shown in Figure S4, indicated the sequencing depths were adequate to downstream analyze; and, the sequencing reads for soil sample at different depths were comparable, whereas the category of observed OTUs decreased with the increase in depths in the soil profile. In total, the observed OTUs could be classified into 47 phyla, 83 classes, 115 orders, 262 families, and 704 genera. Besides, the alpha-diversity indexes, such as Chao1 index, Pielou’s evenness index, Shannon’s diversity index, and Simpson’s index of diversity, were analyzed in order to examine the soil microbes in different depths based on 97% OTU cluster (Figure 4). There was little difference in the Chao1 index of soil at different depths, which suggested that it was identified a comparable quantity of different individuals in different depths of soil, as shown in Figure 4a. As reflected by Figure 4b, however, Pielou’s evenness index was slightly higher in top soil (1–3 m) than in subsoil (4–10 m). As shown in Table S2, the diversity indexes were impacted by soil properties and the contents of metal(loid)s, indicating environmental conditions, such as pH, TK, CEC, ORP, and Bio-Cu (*p* < 0.05), impacted the soil microbial evenness through limiting the survival of certain microorganisms [27,28]; while, soil microbes adapt to higher metal(loid)s pollution in top soil by changing microbial communities, rather than changing their evenness [29]. Moreover, Shannon’s diversity index and Simpson’s index of diversity exhibited a decreasing trend from 1 m to 10 m of soil depth, which might be ascribed to the decrease in evenness with increase in depth, as shown in Figure 4c,d. In addition, OTU composition and structure exhibited difference in a series of soil depth, as reflected by Venn diagram, ANOSIM, NMDS, and DCA analysis at Figure 5. Venn diagram (Figure 5a) revealed that there were 599 core OTUs among ten depth points, and soils in 1 m–10 m had similar unique OTUs. This suggested that distinct microbial communities existed in different depths of soil, because the quantity of core OTUs was less than that of unique OTUs. ANOSIM analysis (Figure 5b) that was based on the Bray Curtis matrix found that the differences of inter-layers were significantly greater than intra-layers due to *p* = 0.001. NMDS analysis (Figure 5c) that was performed on Bray Curtis dissimilarity at OTU level reflected that the microbial communities of 1 m depth, 2 m depth, and 4 m depth soil layers were grouped separately from other depth soil layers. DCA analysis (Figure 5d) also revealed that the coordinate components (DCA1 and DCA2) could explain 27.84% and 11.12% of the total variations, respectively.

**Figure 6:**
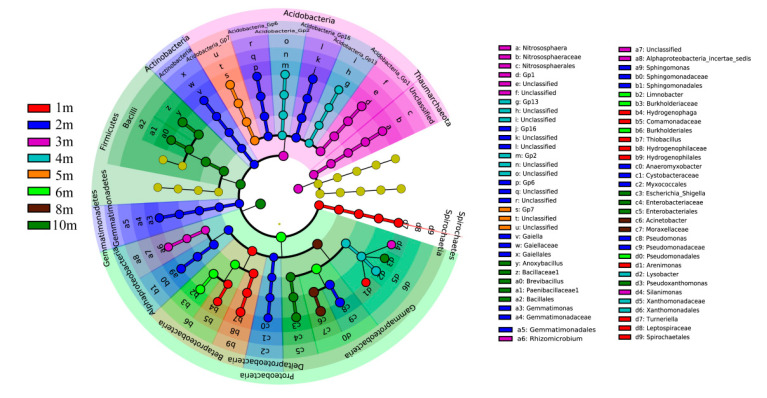
LEfSe analysis of soil samples at different depths (golden circles mean non-significant difference (*p* > 0.05) in abundance among different soil depth layers; other colors mean biomarkers with significant differences (*p* < 0.05) in different soil depth layers).

LEfSe as a reliable method for examining biomarkers in microbiome has been successfully utilized to analyze bacterial biomarkers in metal(loid)s polluted soils [30]. *Acidobacteria* and *Proteobacteria* were generally present under phylum level in soil samples at all depths, As exhibited in Figure 6. *Proteobacteria* accounts for a major proportion of the traditional gram-negative bacteria and plays a crucial act in ecological energy cycle relating to the metabolization of organic and inorganic matters to gain energy [31]. *Acidobacteria* contains an enormous level of morphological, physiological, and metabolic diversity, and both of them are play a crucial role in global soil carbon, nitrogen, and sulfur cycling [32]. However, soil samples at different depths also have their biomarkers in genus level. As seen, *Thiobacillus*, *Nitrososphaera*, and *GP1* were the main biomarkers in top soil (1–3 m). Previous studies have revealed that all of *Thiobacillus*, *Nitrososphaera*, and *GP1* exhibited resistance to metal(loid)s in various environment [33,34,35], which suggested their adaptations to the long-term pollution of high-concentration metal(loid)s. The prominent biomarkers in subsoil (4–10 m) were *Limnobacter*, *Anoxybacillus*, and *Acinetobacter*. Among them, the genus *Anoxybacillus* is a relatively new genus of gram-positive bacteria; and, biochemical and genomic researches found *Anoxybacillus* genus could act as important role in soil carbon recycling in extreme anaerobic environment [36]. This suggested that, although the concentration of metal(loid)s in the subsoil was low, the extreme environment below affected the community structure of microorganisms; and, environmental conditions, including pH, nutrient, and oxygen content, might has selective pressure on soil microbial community.

**Figure 7:**
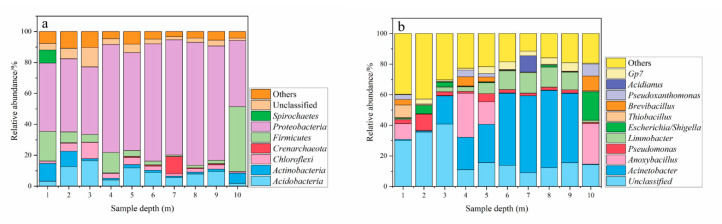
Relative abundance (%) of dominant bacteria and archaea in vertical soil samples at phylum level (**a**) and genus level (**b**).

Besides, the relative abundances of the phyla in different depths of soil is shown in Figure 7a. As seen, *Proteobacteria* (42.8–79.5%), *Acidobacteria* (1.8–16.5%), *Firmicutes* (0.9–41.9%), and *Chloroflexi* (1.0–10.6%) were dominant phyla with high relative abundances in all of the soil sample, which was consistent with the results shown in LEfSe analysis. These predominant phyla also obtained in other metal(loid)s contaminated soil, such as paddy soil, mining sites, and sediment [37,38,39,40], which indicated these phyla might be closely related to metal(loid)s-contaminated soil. For example, Liu et al. also found that *Proteobacteria* (33.0–96.7%), *Actinobacteria* (0.3–6.9%), *Firmicutes* (0.0–5.8%), and *Chloroflexi* (0.0–13.1%) dominated the indigenous soil microbial communities at a chromium salt factory [41]. Except *Proteobacteria* and *Acidobacteria* play a key role in global ecological function on soil energy cycle, the subordinate dominant phyla of *Firmicutes* and *Chloroflexi* were also reported to participate in organic and mineral composition cycles [42,43]. This indicated that these dominant phyla had high metabolic activity and exhibited well adaptability to the metal(loid)s-containing living surroundings. However, for the relative abundance in different depths of soil in this study, the proportion of individuals in different depths existed different. This could demonstrate different in Pielou’s evenness index, as exhibited at Figure 4b. The relative abundance *Proteobacteria* in 1–3 m maintained at about 40%, while it increased to about 70%. Nevertheless, the relative abundance of certain phyla decreased with increase in depth. For example, *Actinobacteria* decreased from about 10% in 1–3 m to 1% in 4–10 m; *Bacteroidetes* decreased from about 3% in 1–3 m to 1% in 4–10 m. This suggested that metal(loid)s pollution and local environmental conditions, including pH, nutrient, and oxygen content, might have selective pressure on soil microbial community [44,45]. At the genus level *Acinetobacter*, *Pseudomonas*, and *Gp7* were dominant genus in soil sample, as reflected by Figure 7b. *Acinetobacter* was the dominant genus in soil samples in 4–10 m and the maximum relative abundance value reached 50%. This might be attributed to the high concentration metal(loid)s pressure in top limiting the growth of this microbe. *Pseudomonas* maintained similar relative abundance in all soil samples, which might due to it as one of the most diverse genera with ubiquitous and versatile microorganisms presenting various metabolic capabilities [46]. In comparison to topsoil, *Gp7* was enriched in subsoil, likely because it prefers to grow in an environment with nutrition deficiency [47].

### 3.3. Relationship between the Environmental Variables and Microbial Communities

**Figure 8:**
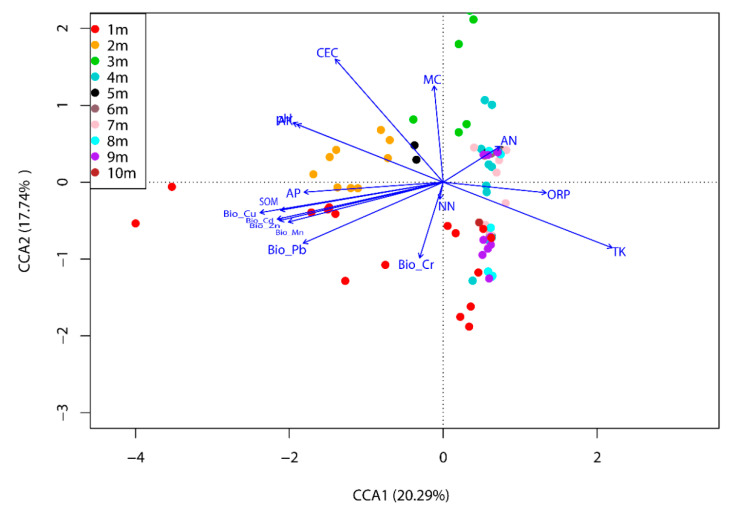
Ordination diagrams from CCA of environmental conditions with overall microbial data.

CCA, as one of Multivariate ordination methods, was used to analyze the significant environmental variables (i.e., pH, ORP, MC, SOM, AN, NN, AP, AK, TK, CEC, and bioavailable metal(loid)s), which influence the microbial community composition, based on OTUs -level data in this study. The CCA method was performed because the length of first DCA axis was more than four SD, suggesting a unimodal ordination technique, as exhibited by Figure 8. As seen, the weights of the community variations explained for the first axis and the second axis were 20.29% and 17.74%, respectively. The relationship between the environmental variables and the microbial community was significant due to *p* < 0.001 in the Monte Carlo permutation test. The results showed that the environmental parameters, such as TK, SOM, ORP, pH, AN, Bio-Cu, Bio-Cd, and Bio-Pb, had a great effect on the abundance and composition of the microbial community (0.001 < *p* < 0.01). Among these parameters, TK, ORP, and AN corresponded more to the subsoil (4–10 m) microbial community, while other remarkable environmental factors were relative to microbial community in the surface soil (1–3 m). Besides, three phyla identified in microbial communities, *Crenarchaeota*, *Gemmatimonadetes*, and *Verrucomicrobia*, exhibited significant positive correlation with pH, and *Firmicutes* showed significant negative correlation with pH, as shown in the Pearson correlation between phylum/genus abundance and soil environmental variables (Tables S3 and S4). As genus leval, *Limnobacter* and *Acinetobacter* also showed significant negative correlation with pH. Indeed, some previous researches had revealed that soil pH value could impact the soil microbial communities and activities through changing chemical forms, contents, and bioavailability of substrates, and then affect microbial cell growth and activity, which results in strong selective pressure for the microbial community structure [41,48]. SOM is related to many essential ecosystem functions and it can remain soil structure, bind mineral particles, and enhance water-holding capacity; in addition, SOM is also a dynamic store of nutrients that cycle to bioavailable forms [48]. Some previous studies also demonstrated that SOM could participate in metal(loid) adsorption, complexation, and precipitation processes, leading to the reduction of the metal(loid) mobility and bioavailability for soil microorganisms [2]. Thus, in this study, some soil microorganisms (*Actinobacteria*, *Bacteroidetes*, and *Gemmatimonadetes* at the phylum level; *Hydrogenophaga* and *Thiobacillus* at the genus level) were significantly affected by SOM. As shift in soil ORP conditions, soil microorganisms can switch to using alternative electron acceptors, such as nitrate, iron, and sulfate, and, thus, changing the microbial metabolic activity and structure in the soil [49]. Therefore, *Limnobacter* and *Acinetobacter* at the genus level were significantly relative to soil ORP. TK and AN were important nutrients for soil biota [50]. Accordingly, Genus *Limnobacter* and *Acinetobacter* were significantly positively related to these nutrients in this research. 

Bioavailable metal(loid)s, including Bio-Cu, Bio-Cd, and Bio-Pb, significantly influenced the microbial community in soil samples. Phylum *Actinobacteria* and *Gemmatimonadetes*, and genus *Hydrogenophaga* and *Thiobacillus* were positive relative to Bio-Cu, Bio-Cd, and Bio-Pb, as exhibited in Tables S3 and S4. In previous studies, El Baz et al. found Actinobacteria that were isolated from abandoned mining areas exhibited a strong ability to resistance and accumulation for Cu, Cd, and Pb [51]. He et al. found that *Gemmatimonadetes* was one of the dominant bacterial phyla in the mixed heavy metal (Cd, Pb, and Zn) polluted paddy fields [52]. *Hydrogenophaga* was a common bacterium flora in contaminated environment, and it needed to obtain their energy through denitrification. Additionally, Liu et al. found it was one of typical denitrifying bacterium in alkaline copper mine drainage [53]. Besides, the genus Thiobacillus are chemolithotrophic and acidophilic and it can oxidize the reduced sulfur or ferrous ion and then generate acid that is favorable for metal removal. This genus is widely used to metal extraction from low-grade ores and mineral concentrates [54]. These indicated that metal(loid)s, including Cu, Cd, and Pb, acted as important environmental factors in the selection of soil microorganism, leading to affecting the diversity and composition of soil microbial community in this study [55]. Moreover, soil microbial communities in vertical soil profile could be impacted by the combined soil factors. The main reasons were that environmental factors, such as TK, SOM, and other nutrients, were generally needed in microbial survival; besides, metal(loid)s pollution, such as As, Mn, and Cd, led stress to microbial survival. Some researches revealed that the soil microbial community was driven by a combination of multiple factors, namely the soil physicochemical characteristics and effects of toxic metal(loid) contaminants [39,56,57]. In addition, the physicochemical characteristics of soil could decrease the toxicity of metal(loid)s, due to TK and SOM being able to provide substrate and nutrients for the growth of microorganisms and then effectively decrease the stress caused by metal(loid)s [58]. For example, some studies found that the nutrient conditions, such as organic matter and CEC, had an important effect on the toxicity of Cu [59]. Environmental conditions including nutrient availability, organic matter, and metal(loid)s bioavailability were also generally associated with soil pH and ORP and the change in pH and ORP could result in changes in the microbial community structure [60,61].

## 4. Conclusions

In the present study, the results revealed that most metal(loid)s mainly occurred at 1–2 m in the depth of soil and Mn, Pb, Zn, and Cu were relative abundant in the vertical soil profile, whereas As, Cd, Pb, and Mn were the risk metal(loid) pollutants. The bioavailability of As, Cr, and Cu was comparably low. Besides, the microbial community diversity decreased with an increase in soil depth due to the change in evenness. The dominant soil microbial community at the phylum level was *Proteobacteria*, *Acidobacteria*, *Firmicutes*, and *Chloroflexi* and at genus level was *Acinetobacter*, *Pseudomonas*, and *Gp7*. These microbes might play an important role in soil ecological function and the biogeochemistry of metal(loid)s. Environmental parameters, including TK, SOM, ORP, pH, AN, Bio-Cu, Bio-Cd, and Bio-Pb had great influence on composition and diversity of the microbial community in soil.

## Figures and Tables

**Table 1:** Physicochemical properties of studied soil samples.

Depth (m)	pH	ORP (mV)	Sand (%)	Silt (%)	Clay (%)	MC (%)	SOM (%)	AN (mg/kg)	NN (mg/kg)	AP (mg/kg)	AK (mg/kg)	TK (mg/kg)	CEC (cmol(+)/kg)
1	7.5 ± 0.3 ^a^	205 ± 38 ^b^	54.3 ± 5.9 ^a^	26.6 ± 1.6 ^a^	19.1 ± 6.7 ^b^	19.4 ± 2.9 ^a^	6.5 ± 1.4 ^a^	45.2 ± 18.1 ^a^	51.6 ± 2.3 ^a^	20.6 ± 9 ^a^	410 ± 1.8 ^a^	9157 ± 2588 ^d^	6 ± 1.3 ^a,b,c^
2	7.5 ± 0 ^a^	229 ± 13 ^a,b^	19.8 ± 7.3 ^b^	35.4 ± 10.4 ^a^	44.9 ± 11.5 ^a,b^	21.9 ± 2.4 ^a^	2.2 ± 0.6 ^b^	55.7 ± 23.5 ^a^	45.7 ± 2.2 ^a^	9.1 ± 5.7 ^a,b^	246.7 ± 132.7 ^a,b^	15709 ± 427 ^c^	8.2 ± 0.5 ^a^
3	7.3 ± 0.1 ^a^	243 ± 6 ^a,b^	13.4 ± 2.6 ^b^	35 ± 17.4 ^a^	51.6 ± 17.1 ^a^	23 ± 0.9 ^a^	0.8 ± 0.2 ^b^	56.6 ± 15.2 ^a^	46.3 ± 2.5 ^a^	4.2 ± 2 ^a,b^	238.4 ± 137 ^a,b^	18444 ± 589 ^c^	7.9 ± 1.4 ^a,b^
4	6.6 ± 0.1 ^a^	280 ± 33 ^a,b^	14.8 ± 4.2 ^b^	49.3 ± 2.5 ^a^	35.9 ± 6.6 ^a,b^	22.9 ± 1.8 ^a^	0.9 ± 0.8 ^b^	65.7 ± 17.2 ^a^	48.6 ± 4.1 ^a^	5.4 ± 5.6 ^a,b^	162.2 ± 95.1 ^a,b^	26331 ± 2218 ^b^	5.1 ± 1.1 ^b,c,d^
5	5 ± 0.4 ^b^	301 ± 44 ^a,b^	21 ± 6.7 ^b^	54.2 ± 9.2 ^a^	24.8 ± 4 ^b^	21.9 ± 0.8 ^a^	0.3 ± 0.1 ^b^	52.8 ± 9.5 ^a^	48.9 ± 4.5 ^a^	6.7 ± 7.7 ^a,b^	125.6 ± 59.3 ^b^	28155 ± 1581 ^a,b^	3.4 ± 0.6 ^c,d^
6	4.5 ± 0.1 ^b^	323 ± 36 ^a^	26.1 ± 4.6 ^b^	47 ± 7.1 ^a^	26.9 ± 2.5 ^a,b^	20.6 ± 0.8 ^a^	0.4 ± 0.1 ^b^	59.2 ± 15.3 ^a^	55.2 ± 9.9 ^a^	3.6 ± 3.7 ^a,b^	80.8 ± 16.3 ^b^	28456 ± 1572 ^a,b^	3.6 ± 0.4 ^c,d^
7	4.5 ± 0.2 ^b^	306 ± 15 ^a,b^	27.1 ± 6.9 ^b^	46.5 ± 5.2 ^a^	26.4 ± 4.2 ^a,b^	20.7 ± 1.4 ^a^	0.3 ± 0 ^b^	58.3 ± 10.4 ^a^	50.2 ± 3.2 ^a^	2.4 ± 2.5 ^b^	77.2 ± 14.2 ^b^	29470 ± 1267 ^a,b^	3 ± 0.5 ^d^
8	4.9 ± 0.4 ^b^	290 ± 8 ^a,b^	23.9 ± 2.7 ^b^	51.7 ± 2.8 ^a^	24.4 ± 0.9 ^b^	20.4 ± 1 ^a^	0.5 ± 0.3 ^b^	73.6 ± 6.5 ^a^	45.9 ± 1.6 ^a^	2.7 ± 1.5 ^b^	66.8 ± 21 ^b^	31158 ± 547 ^a,b^	3.3 ± 0.5 ^c,d^
9	4.8 ± 0.4 ^b^	278 ± 16 ^a,b^	26.3 ± 8.3 ^b^	45.1 ± 4.3 ^a^	28.6 ± 4 ^a,b^	18.7 ± 1.8 ^a^	0.5 ± 0.1 ^b^	70 ± 11.5 ^a^	47.8 ± 9.2 ^a^	4.7 ± 3.7 ^a,b^	77 ± 8.5 ^b^	32678 ± 497 ^a^	3.1 ± 0.3 ^d^
10	4.6 ± 0.6 ^b^	272 ± 42 ^a,b^	26.6 ± 2.4 ^b^	49.4 ± 2.7 ^a^	24 ± 0.6 ^b^	17.9 ± 0.9 ^a^	0.8 ± 0.6 ^b^	48.9 ± 1.5 ^a^	47.2 ± 5.9 ^a^	3 ± 1.8 ^b^	90.1 ± 10.8 ^b^	31309 ± 1184 ^a,b^	3.2 ± 0.5 ^c,d^

Different letters (a, b, c and d) in the same column reflect significant differences among samples at the level of *p* < 0.05 (based on the Duncan multiple range test).

## Supplementary Materials

The following are available online at https://www.mdpi.com/1660-4601/17/19/7101/s1, Figure S1: Soil samples in different depth of soil profile collected at three holes; Figure S2: The background values of metal(loid)s in this site; Figure S3: Speciation distributions of As and Cr in soil profile at different depths; Figure S4: Rarefaction curve of 16S rRNA sequencing in soil samples at different depths; Table S1: Distribution of metal(loid) bioavailable fractions in different soil depths; Table S2: Pearson correlation coefficient of soil environmental variables with microbial diversity index; Table S3: Pearson correlation between phylum abundance and soil physicochemical parameters and bioavailable metal(loid)s; Table S4: Pearson correlation between genus abundance and soil physicochemical parameters and bioavailable metal(loid)s.
