# Supplementary material for: Spatial Distribution of Toxic Metal(loid)s and Microbial Community Analysis in Soil Vertical Profile at an Abandoned Nonferrous Metal Smelting Site
Source: Int J Environ Res Public Health. 2020 Sep 28;17(19):7101. doi: 10.3390/ijerph17197101 (PMC7579518; doi:10.3390/ijerph17197101)
Supplement: Supplementary file 1 [file ijerph-17-07101-s001.pdf]

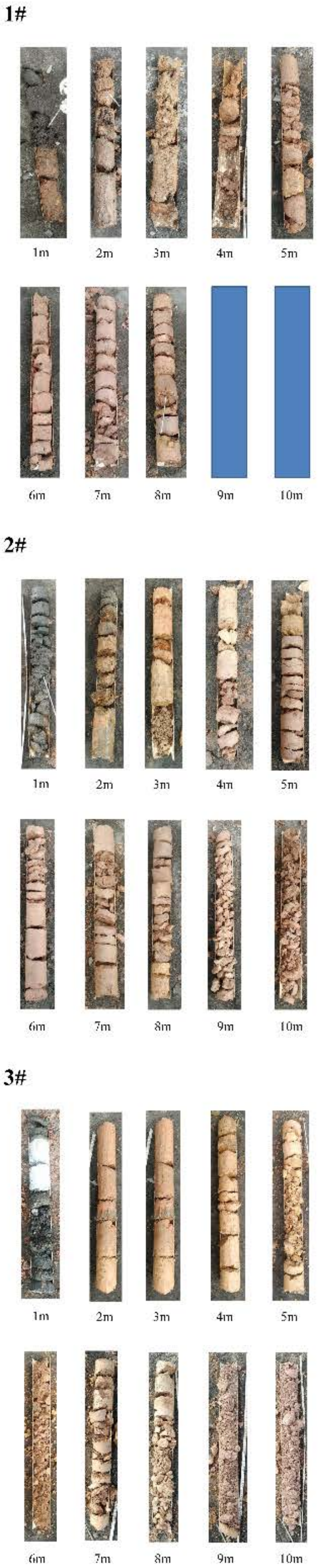

Figure S1. Soil samples in different depth of soil profile collected at three holes.

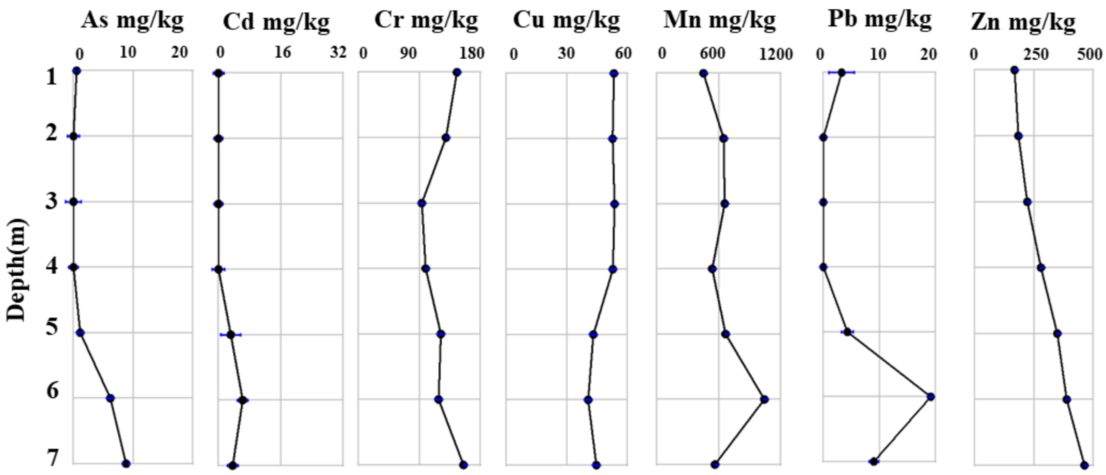

Figure S2. The background values of metal(loid)s in this site.

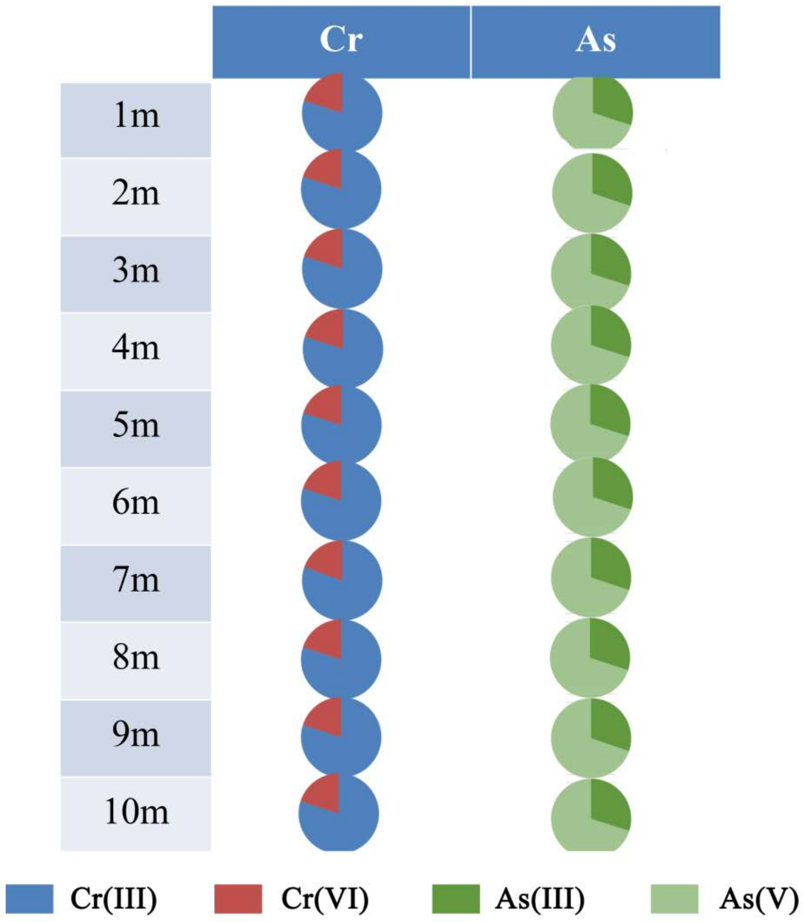

Figure S3. Speciation distributions of As and Cr in soil profile at different depths.

Cr(VI) determination: Alkaline digestion-agents (sodium hydroxide and sodium carbonate) were added to digest the soil, and the supernatant was examined via a UV–vis spectrophotometer (PerkinElmer LAMBDA 950, USA) at wavelength of 540 nm using the 1.5-diphenylcarbazide method.

Cr(III) determination: Adding strong acid to dissolve the soil, the Cr(III) was oxidized to the Cr(VI). Then the supernatant was analyzed similar with Cr(VI) determination. When previous Cr(VI) content was subtracted, the Cr(III) content was obtained.

As(III) determination: The content of As(III) was determined by atomic fluorescence spectrometry (Beijing Titan Instruments Co. Ltd., AFS 9130, China) after the addition of extractants (phosphoric acid and ascorbic acid) to the soil.

As(V) determination: Adding reductants (thiourea solution and ascorbic acid), the As(V) was reduced to As(III). Then the supernatant was analyzed similar with As(III) determination. When previous As(III) content was subtracted, the As(V) content was obtained.

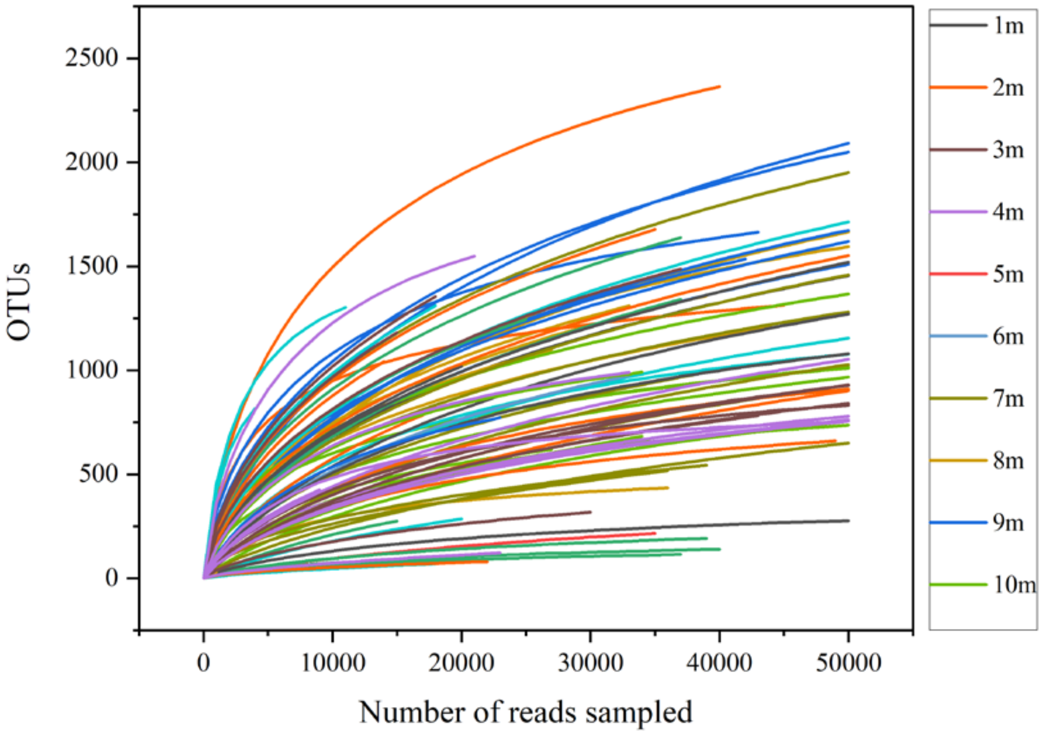

Figure S4. Rarefaction curve of 16S rRNA sequencing in soil samples at different depths.

Table S1. Concentration of metal(loid) bioavailable fractions (mg/kg; mean ± SD) in different soil depths (The detection limits of Cu and As are 0.0054 and 0.053 mg/kg respectively)

Element	1 m	2 m	3 m	4 m	5 m	6 m	7 m	8 m	9 m	10 m
Cd	362±1	47.8±8.85	0.74±15.5	1.25±14.3	0.31±13.9	0.32±8.42	0.33±4.54	0.13±8.06	1.07±5.38	0.43±5.13
Cu	95.3±2.18	47.8±2.71	ND	ND	ND	ND	ND	ND	ND	ND
Mn	516±5	151±12	51.5±5.73	36.5±0.58	45.2±8.82	45.9±0.76	34.4±0.24	39.3±2.04	77.8±1.67	36.4±1.71
Pb	1035±3	79.8±8.6	31.4±5.18	115±2	150±2	118±1	119±2	147±1	164±1	112±1
Zn	25180±4	1889±13	59.3±10.4	82.1±16	27.6±8.55	27.1±7.86	26.9±1.91	13.7±1.89	84±5.47	32.9±6.12
As	ND	ND	ND	ND	ND	ND	ND	ND	ND	ND
Cr	0.345±8.6	0.06±18.5	0.01±127	0.09±19.1	0.08±4.33	0.08±31.5	0.08±8.56	0.16±9.9	0.54±4.65	0.09±8.31

Table S2. Pearson correlation coefficient of soil environmental variables with microbial diversity index (Green cells indicate the *p* values are less than 0.05; 0 means the *p* value was less than 0.001).

Variables	Shannon's diversity		Simpson's index of diversity		Pielou's evenness		Chao1	
	Pearson	*P*	Pearson	*P*	Pearson	*P*	Pearson	*P*
pH	0.425	0	0.487	0	0.329	0.002	-0.338	0.001
ORP	-0.243	0.021	-0.366	0	-0.213	0.043	0.348	0.001
MC	0.236	0.025	0.208	0.049	0.252	0.016	-0.095	0.373
AK	0.206	0.052	0.328	0.002	0.284	0.007	-0.451	0
TK	-0.394	0	-0.536	0	-0.394	0	0.378	0
CEC	0.474	0	0.595	0	0.483	0	-0.355	0.001
AP	0.197	0.063	0.258	0.014	0.235	0.026	-0.115	0.282
AN	0.003	0.976	0.014	0.894	0.017	0.875	0.362	0
NN	-0.059	0.579	-0.065	0.546	-0.001	0.996	0.114	0.284
SOM	0.117	0.272	0.298	0.004	0.14	0.187	-0.33	0.001
Sand	-0.011	0.918	0.065	0.545	-0.014	0.897	-0.054	0.614
Silt	-0.255	0.015	-0.404	0	-0.276	0.008	0.322	0.002
Clay	0.248	0.019	0.312	0.003	0.27	0.01	-0.246	0.019
Bio-Cd	0.121	0.257	0.253	0.016	0.125	0.242	-0.3	0.004
Bio-Cu	0.281	0.007	0.394	0	0.245	0.02	-0.234	0.027
Bio-Mn	0.182	0.087	0.306	0.003	0.168	0.114	-0.268	0.011
Bio-Pb	0.022	0.837	0.139	0.191	0.028	0.791	-0.256	0.015
Bio-Zn	0.085	0.428	0.22	0.038	0.096	0.368	-0.316	0.002
Bio-Cr	-0.021	0.843	-0.003	0.976	-0.088	0.409	0.197	0.063

Table S3. Pearson correlation between phylum abundance and soil physicochemical parameters and bioavailable metal(loid)s (Green cells indicate the *p* values are less than 0.05; 0 means the *p* value was less than 0.001).

Phylum	pH		ORP		MC		AK		TK		CEC		AP		AN		NN		SOM		Bio-Cd		Bio-Cu		Bio-Mn		Bio-Pb		Bio-Zn		Bio-Cr	
	Pearson	*P*	Pearson	*P*	Pearson	*P*	Pearson	*P*	Pearson	*P*	Pearson	*P*	Pearson	*P*	Pearson	*P*	Pearson	*P*	Pearson	*P*	Pearson	*P*	Pearson	*P*	Pearson	*P*	Pearson	*P*	Pearson	*P*	Pearson	*P*
Acidobacteria	-0.119	0.264	0.123	0.248	-0.085	0.425	0.065	0.54	0.058	0.586	-0.089	0.406	0.09	0.4	-0.191	0.071	-0.031	0.773	0.077	0.469	0.061	0.571	0.007	0.948	0.057	0.591	0.088	0.408	0.075	0.48	0.02	0.851
Actinobacteria	0.341	0.001	-0.28	0.008	-0.025	0.814	0.358	0.001	-0.365	0	0.236	0.025	0.401	0	-0.206	0.051	-0.041	0.7	0.475	0	0.43	0	0.478	0	0.456	0	0.389	0	0.409	0	0.265	0.012
Bacteroidetes	0.316	0.002	-0.254	0.016	0.041	0.702	0.432	0	-0.425	0	0.256	0.015	0.452	0	-0.303	0.004	-0.001	0.995	0.439	0	0.42	0	0.375	0	0.418	0	0.388	0	0.422	0	0.056	0.6
Chloroflexi	0.352	0.001	-0.155	0.145	0.11	0.304	0.459	0	-0.393	0	0.323	0.002	0.354	0.001	-0.141	0.186	0.064	0.552	0.31	0.003	0.385	0	0.318	0.002	0.364	0	0.354	0.001	0.381	0	0.063	0.556
Crenarchaeota	0.442	0	-0.299	0.004	0.111	0.299	0.276	0.008	-0.382	0	0.458	0	0.126	0.237	0.174	0.101	-0.043	0.685	0.244	0.021	0.086	0.419	0.351	0.001	0.143	0.18	-0.051	0.632	0.044	0.683	-0.148	0.165
Euryarchaeota	0.223	0.035	-0.076	0.479	-0.049	0.645	0.186	0.079	-0.224	0.034	0.172	0.106	0.227	0.031	-0.234	0.026	0.043	0.686	0.196	0.064	0.213	0.044	0.243	0.021	0.237	0.025	0.183	0.084	0.206	0.052	0.05	0.642
Firmicutes	-0.595	0	0.341	0.001	-0.198	0.061	-0.511	0	0.601	0	-0.614	0	-0.384	0	0.073	0.492	0.069	0.518	-0.451	0	-0.359	0.001	-0.508	0	-0.396	0	-0.23	0.03	-0.325	0.002	0.083	0.434
Gemmatimonadetes	0.46	0	-0.255	0.015	-0.293	0.005	0.427	0	-0.473	0	0.251	0.017	0.306	0.003	-0.192	0.07	0.101	0.343	0.499	0	0.588	0	0.545	0	0.577	0	0.553	0	0.592	0	0.166	0.118
Proteobacteria	0.36	0	-0.174	0.102	0.33	0.001	0.273	0.009	-0.319	0.002	0.431	0	0.128	0.231	-0.141	0.185	-0.063	0.556	0.077	0.469	0.082	0.442	0.092	0.39	0.093	0.382	0.015	0.89	0.076	0.476	-0.16	0.133
Spirochaetes	0.38	0	-0.25	0.018	0.085	0.424	0.235	0.026	-0.297	0.004	0.396	0	0.082	0.444	0.146	0.168	-0.037	0.732	0.176	0.097	0.009	0.932	0.261	0.013	0.065	0.54	-0.115	0.281	-0.032	0.763	-0.16	0.133
Thaumarchaeota	-0.131	0.219	0.384	0	-0.026	0.809	-0.144	0.177	0.172	0.105	-0.15	0.159	-0.01	0.929	0.095	0.373	0.086	0.421	-0.176	0.098	-0.134	0.001	-0.172	0.218	-0.136	0.091	-0.092	0	-0.129	0.096	0.065	0
Unclassified	0.344	0.001	-0.143	0.179	0.042	0.691	0.417	0	-0.441	0	0.168	0.115	0.465	0	-0.291	0.005	0.086	0.422	0.452	0	0.534	0	0.466	0	0.509	0	0.515	0	0.519	0	0.178	0.094
Verrucomicrobia	0.484	0	-0.358	0.001	0.196	0.064	0.38	0	-0.466	0	0.551	0	0.223	0.034	0.161	0.13	-0.049	0.645	0.283	0.007	0.15	0.116	0.391	0.002	0.199	0.333	0.004	0.24	0.107	0	-0.162	0.839

Table S4. Pearson correlation between genus abundance and soil physicochemical parameters and bioavailable metal(loid)s (Green cells indicate the *p* values are less than 0.05; 0 means the *p* value was less than 0.001).

Genus	pH		ORP		MC		AK		TK		CEC		AP		AN		NN		SOM		Bio-Cd		Bio-Cr		Bio-Cu		Bio-Mn		Bio-Pb		Bio-Zn	
	Pearson	*P*	Pearson	*P*	Pearson	*P*	Pearson	*P*	Pearson	*P*	Pearson	*P*	Pearson	*P*	Pearson	*P*	Pearson	*P*	Pearson	*P*	Pearson	*P*	Pearson	*P*	Pearson	*P*	Pearson	*P*	Pearson	*P*	Pearson	*P*
Anoxybacillus	-0.066	0.537	0.046	0.671	-0.391	0	-0.034	0.751	0.144	0.179	-0.108	0.312	0.054	0.613	-0.109	0.307	-0.109	0.308	-0.049	0.645	0.153	0.15	-0.034	0.751	0.101	0.342	0.116	0.276	0.155	0.145	0.163	0.124
Limnobacter	-0.564	0	0.535	0	-0.28	0.008	-0.41	0	0.552	0	-0.583	0	-0.348	0.001	0.226	0.033	-0.005	0.963	-0.468	0	-0.372	0	0.158	0.138	-0.468	0	-0.392	0	-0.245	0.02	-0.35	0.001
Sphingobacterium	0.115	0.284	-0.164	0.126	-0.186	0.081	0.086	0.423	-0.146	0.173	0.24	0.023	0.151	0.158	-0.207	0.051	-0.106	0.323	0.137	0.2	0.116	0.276	-0.069	0.518	0.097	0.361	0.096	0.371	0.108	0.31	0.12	0.261
Acidovorax	-0.151	0.157	0.062	0.566	-0.49	0	0.073	0.497	0.087	0.415	-0.255	0.016	0.075	0.486	-0.297	0.005	0.069	0.519	-0.033	0.756	0.258	0.014	0.035	0.745	0.189	0.075	0.22	0.037	0.277	0.008	0.27	0.01
Acinetobacter	-0.412	0	0.428	0	-0.056	0.602	-0.329	0.002	0.375	0	-0.399	0	-0.459	0	0.178	0.096	-0.109	0.308	-0.049	0.645	-0.424	0	0.099	0.353	-0.522	0	-0.445	0	-0.311	0.003	-0.402	0
Hydrogenophaga	0.193	0.071	-0.318	0.002	-0.303	0.004	0.201	0.059	-0.086	0.421	0.043	0.69	0.223	0.035	-0.171	0.11	-0.005	0.963	-0.468	0	0.438	0	0.159	0.135	0.407	0	0.439	0	0.439	0	0.447	0
Nitrososphaera	0.21	0.048	-0.155	0.146	0.366	0	-0.01	0.928	-0.137	0.199	0.259	0.014	-0.044	0.685	0.159	0.136	-0.106	0.323	0.137	0.2	-0.092	0.39	-0.146	0.17	-0.094	0.381	-0.087	0.416	-0.13	0.221	-0.09	0.398
Pseudomonas	0.122	0.255	-0.095	0.376	0.152	0.156	-0.01	0.928	-0.173	0.105	0.138	0.199	-0.176	0.099	-0.002	0.987	0.069	0.519	-0.033	0.756	0.007	0.945	-0.128	0.228	0.151	0.154	0.062	0.559	-0.059	0.578	-0.015	0.89
Brevibacillus	-0.037	0.734	-0.06	0.579	-0.366	0	-0.004	0.967	0.151	0.158	-0.089	0.406	0.043	0.688	-0.092	0.392	-0.134	0.212	0.001	0.991	0.155	0.146	-0.032	0.762	0.103	0.336	0.118	0.268	0.157	0.14	0.165	0.121
Sphingomonas	0.229	0.031	-0.289	0.006	0.104	0.333	-0.002	0.988	-0.156	0.144	0.127	0.237	-0.013	0.902	0.017	0.872	0.01	0.928	0.144	0.179	0.012	0.909	-0.089	0.405	0.131	0.219	0.034	0.751	-0.041	0.701	-0.004	0.967
Methylobacterium	0.076	0.481	-0.1	0.351	-0.188	0.077	-0.126	0.241	0.028	0.795	-0.107	0.32	0.046	0.669	0.028	0.793	-0.013	0.902	0.004	0.973	0.144	0.176	-0.042	0.694	0.266	0.011	0.19	0.072	0.063	0.556	0.125	0.239
Thiobacillus	0.042	0.695	0.001	0.995	-0.086	0.421	-0.079	0.46	-0.079	0.46	-0.07	0.512	-0.035	0.746	0.058	0.592	0.035	0.742	0.067	0.534	0.624	0	0.252	0.017	0.558	0	0.594	0	0.617	0	0.602	0
Escherichia/Shigella	0.031	0.773	-0.159	0.136	-0.033	0.761	0.11	0.305	0.11	0.305	0.072	0.505	0.1	0.352	0.071	0.51	-0.26	0.014	0.031	0.774	-0.08	0.453	-0.144	0.175	-0.035	0.74	-0.084	0.432	-0.106	0.319	-0.087	0.416
Gp7	-0.266	0.012	-0.035	0.742	-0.351	0.001	0.247	0.02	0.247	0.02	-0.264	0.012	-0.201	0.059	0.194	0.068	-0.056	0.6	-0.312	0.003	-0.366	0	0.154	0.147	-0.297	0.004	-0.317	0.002	-0.322	0.002	-0.373	0
Pseudoxanthomonas	0.09	0.401	-0.209	0.049	0.264	0.012	-0.11	0.304	-0.11	0.304	0.193	0.07	0.146	0.173	-0.154	0.149	0.03	0.777	0.127	0.236	0.185	0.081	-0.021	0.846	0.129	0.224	0.147	0.167	0.186	0.08	0.195	0.065
Unclassified	0.402	0	0.341	0.001	0.188	0.078	-0.402	0	-0.402	0	0.372	0	0.316	0.003	0.042	0.693	0.003	0.975	0.322	0.002	0.189	0.074	-0.088	0.408	0.29	0.005	0.235	0.026	0.086	0.419	0.172	0.104
